# Adipose Tissue Quality in Aging: How Structural and Functional Aspects of Adipose Tissue Impact Skeletal Muscle Quality

**DOI:** 10.3390/nu11112553

**Published:** 2019-10-23

**Authors:** Flavia G. De Carvalho, Jamie N. Justice, Ellen C. de Freitas, Erin E. Kershaw, Lauren M. Sparks

**Affiliations:** 1 Translational Research Institute, Advent Health, 301 East Princeton Street, Orlando, FL 32804, USA; flaviagiolo@gmail.com; 2 School of Physical Education and Sport of Ribeirao Preto, University of Sao Paulo, Avenida Bandeirantes 3900, Ribeirao Preto, SP, 14040-907, Brazil; ellenfreitas@usp.br; 3 Internal Medicine Section on Gerontology and Geriatric Medicine, Wake Forest School of Medicine, 1 Medical Center Blvd, Winston-Salem, NC 27157, USA; jnjustic@wakehealth.edu; 4 Division of Endocrinology and Metabolism, Department of Medicine, University of Pittsburgh, Pittsburgh, PA 15260, USA; kershawe@pitt.edu

**Keywords:** adipose tissue, skeletal muscle, aging, mobility, senescence, inflammation, secretory

## Abstract

The interplay between adipose tissue and skeletal muscle and the impact on mobility and aging remain enigmatic. The progressive decline in mobility promoted by aging has been previously attributed to the loss of skeletal mass and function and more recently linked to changes in body fat composition and quantity. Regardless of body size, visceral and intermuscular adipose depots increase with aging and are associated with adverse health outcomes. However, the quality of adipose tissue, in particular abdominal subcutaneous as it is the largest depot, likely plays a significant role in aging outcomes, such as mobility decline, though its communication with other tissues such as skeletal muscle. In this review, we discuss the age-associated development of a pro-inflammatory profile, cellular senescence, and metabolic inflexibility in abdominal subcutaneous adipose tissue. Collectively, these facets of adipose tissue quality influence its secretory profile and crosstalk with skeletal muscle and likely contribute to the development of muscle atrophy and disability. Therefore, the identification of the key structural and functional components of adipose tissue quality—including necrosis, senescence, inflammation, self-renewal, metabolic flexibility—and adipose tissue-secreted proteins that influence mobility via direct effects on skeletal muscle are necessary to prevent morbidity/mortality in the aging population.

## 1. Introduction

Addressing the needs of the rapidly expanding aging population has become one of most daunting challenges of our time. The number of people ≥65 years of age is expected to more than double to 88.5 million between 2010 and 2050, representing more than 20% of the US population by 2030 [1]. A staggering 35% of people with an age ≥65 years suffer from physical disability, with more than 17% unable to walk a quarter mile [2]. This aging-related mobility disability is associated with greater loss of activities of daily living, hospitalizations/institutionalization, heath care costs and ultimately, mortality [3,4,5].

Substantial interest has focused on identifying links between biological drivers of aging and functional decline, including factors regulating inter-cellular and inter-organ communication that contribute to system-wide pathophysiology and lead to functional decline and disability. Of relevance is the contribution of adipose tissue to age-related mobility disability and, in particular, to skeletal muscle quality and function which determine force production and motion during aging.

Mobility disability in aging has largely been attributed to skeletal muscle characteristics; however, adipose tissue is also likely to play a critical role in this process. Adipose tissue has multiple characteristics that may contribute to muscle health and disease. Adipose tissue mass is correlated with skeletal muscle function and mobility, such that adipose tissue deposition and loss of muscle mass and strength occur simultaneously with aging and in other muscle-related diseases [6,7,8]. In addition to mass or quantity, the quality of adipose tissue, namely cellular composition, fatty acid storage capacity [9], and secreted factors, is also likely to influence mobility.

Appreciation of the physiological relevance of adipose tissue has radically changed over the past twenty-five years. The adipose organ is comprised of adipose stem cells (ASCs) and adipocytes, as well as a myriad of other cell types including fibroblasts, endothelial cells, and immune cells such as macrophages, dendritic cell, T cells, neutrophils, natural killer (NK) lymphocytes, eosinophils, mast cells, NK-cells, et al. [10]. ASCs are precursor cells that give rise to new adipocytes. Mature adipocytes are the primary cell type responsible for storing energy as fat. Although fat storage was once thought to be the sole function of adipose tissue, it is now known that adipose tissue is a highly active endocrine organ that secretes numerous bioactive substances including adipocyte-secreted proteins/peptides (i.e., adipokines), immune cell-secreted proteins/peptides (i.e., chemokines, cytokines), and bioactive lipids (i.e., lipokines) and communicates with other cells and tissues throughout the body.

Hence, the mass, regional location, structure, function, and cellular composition of adipose tissue can all influence is secretory profile and physiological impact. The discovery of leptin in 1994, a critical hormone in energy balance produced principally by white adipocytes, solidified adipose tissue as an important endocrine organ [11]. In humans, adipose tissue secretory profiles differ by metabolic phenotype (e.g., lean vs. obese) [12], yet the effects of advancing age (i.e., >65 years) on these secretory profiles and their potential role in the aging process have not been addressed in humans [13]. There is some evidence about secreted factors such as a secretory-associated senescent phenotype secreted by senescent cells within the adipose tissue with the ability to reduce expressions of contractile proteins and induce insulin resistance in human muscle cells [14], suggesting a potentially important role for adipose tissue and adipose tissue-secreted factors in mediating declines in muscle function and mobility with aging.

Furthermore, adipocyte lipid turnover is strongly related to conditions with disturbed lipid metabolism such as obesity, dyslipidemia, and insulin resistance [15] that can occur with aging. Previously published studies in humans showed that impairments in metabolic flexibility are linked to adipose tissue inflammation and hypertrophic adipocytes [16,17]. Adipocyte metabolic flexibility refers to the ability of the fat cell to regulate storage and release of fatty acids (FAs) which impacts whole-body physiological homeostasis [17]. Recent evidence suggests that reduced adipocyte metabolic flexibility related to the PNPLA2/ATGL pathway is associated with skeletal muscle dysfunction and mobility decline [18]. Thus, adipose tissue is a highly dynamic organ with the potential to influence muscle function and whole-body metabolism during aging.

The skeletal muscle also secretes peptides and non-peptides (i.e., irisin, meteorin-like 1, myostatin, β-aminoisobutyric acid, myonectin fibroblast growth factor 21, myokines, et al.) that can migrate to peripheral tissues with varying effects [19,20]. For example, myokines can regulate energy balance, glucose, and lipid whole-body metabolism, and also regulate the adipose tissue metabolism [21]. However, in aging, some myokines (e.g., MCP-1, myostatin. TNF-a, IL-6, IL-10, and IL-1b) [22] can negatively impact the adipose tissue by promoting an inflammatory feedback loop that furthers adipose cytokines release (TNF-α, IL-1b, IL-6 and monocyte chemoattractant protein) from adipose tissue macrophages [23,24], leading to immunosenescence and impairments in whole-body metabolism [13]. Therefore, there is a bidirectional effect from skeletal muscle peptides and non-peptides that affect adipose tissue function [21]; however, this review emphasizes how the adipose tissue quality and its secretory profile impacts skeletal muscle function and mobility in aging.

**Figure 1:**
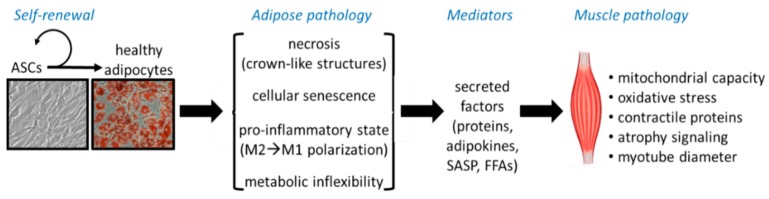
Key structural and functional components of adipose tissue and adipose tissue-secreted factors to key biological properties of skeletal muscle and mobility in aging. Adipose tissue arises from adipose-derived stem cells (ASCs). The ability of ASCs to self-renew is critical for maintenance of healthy adipocytes and adipose tissue. While some ASCs retain the capacity for ongoing regeneration, others change their self-renewal cycle to become mature adipocytes. Mature “healthy” adipocytes have several potential fates that may contribute to “adipose pathology:” cell death (in which cells usually develop necrosis and are engulfed by macrophage, forming crown-like structures), senescence (in which cells cease to divide and enter a quiescent, metabolically inactive state), inflammation (in which inflammatory cells infiltrate adipose tissues and produce inflammatory cytokines), metabolic inflexibility (in which typically hypertrophic cells develop reduced ability to adjust substrate utilization in response to physiological stimuli such as insulin and catecholamines). These adipose “fates,” in turn, influence secretion of bioactive factors that can ultimately mediate inter-organ pathology, including skeletal muscle pathology. SASP, secretory-associated senescent phenotype; FFAs, free fatty acids; M1, macrophage 1; M2, macrophage 2.

Figure 1 summarizes the contributions of key structural and functional components of adipose tissue (regional mass, necrosis, senescence, inflammation, self-renewal, and metabolic flexibility) and adipose tissue-secreted factors to key biological properties of skeletal muscle and mobility outcomes in aging through adipose tissue-skeletal muscle crosstalk.

## 2. Adipose Tissue and Lipid Distribution with Aging

Aging is characterized by changes in adipose tissue distribution. These changes include redistribution of adipose tissue from peripheral subcutaneous locations to intra-abdominal (visceral) and inter-muscular (IMAT) locations, as well as redistribution of lipids from adipose tissue to metabolically-relevant non-adipose tissues such as liver (intrahepatic lipid, IHL) and muscle (intramyocellular lipid, IMCL), resulting in “lipotoxicity” [25,26,27]. Lipotoxicity is the process whereby deleterious lipid species accumulate and cause cellular harm. In skeletal muscle tissue, IMCL can be safely stored as neutral lipids in lipid droplets. The balance between inert storage of IMCL as neutral lipids in lipid droplets and their release for cellular functions (i.e., mitochondrial oxidation for energy or incorporation into cellular membranes) is tightly regulated by a complement of lipases (i.e., ATGL/PNPLAs, LIPE/HSL) and lipid droplet proteins (i.e., Plin5) [25]. Imbalance in this tightly regulated process leads to accumulation of “toxic” lipids such as ceramides and diacylglycerols which have been shown to promote insulin resistance, endoplasmic reticulum (ER) stress, mitochondrial dysfunction and apoptosis [28,29].

Several studies have evaluated the relationship between IMCL and skeletal muscle dysfunction (reduction of muscle fiber size, fiber number and contractility), in particular muscle insulin resistance, while fewer studies have evaluated the relationship between IMCL and skeletal muscle function in aging. Notably, one study comparing IMCL in muscle fibers of young active versus older sedentary adult men found that the older sedentary men had higher lipid content, especially type I fibers, as well as multiple deleterious phenotypes in all fiber types, especially type II fibers. These deleterious phenotypes included lower mitochondrial content and oxidative capacity [30]. The relative contribution of IMCL to these deleterious phenotypes and vice versa is not yes entirely clear, particularly in aging skeletal muscle. However, since this review focuses on the quality and function of adipose tissue and not lipid per se, the role of IMCL in aging will not be further discussed.

An understudied concept is the contribution adipose tissue quality to skeletal muscle function, mobility, and aging. One aspect of adipose tissue quality is total and regional adiposity. Regardless of body size, visceral fat and intermuscular fat (IMAT) increase with age and are associated with impaired single-fiber contractility and lower muscle strength and power [23,31]. A report from the Health ABC Study cohort (1678 men and women aged 70 to 79 years) demonstrated that upper and lower extremity strength and muscle quality decrease with age. The study investigated age-related changes in leg composition (midthigh muscle, subcutaneous fat and intermuscular fat), strength (isokinetic leg muscle torque), and muscle quality (muscle torque/quadriceps cross-sectional area) over 5 years. Regardless of muscle mass and body weight changes and changes in subcutaneous thigh adipose tissue, a progressive muscle weakness and an increase in muscular fat infiltration occurs with aging, resulting in losses of strength and muscle quality was observed [32].

In addition, high and low total adiposity independently contributed to the effects of aging on muscle quality. Another report from the Health ABC Study (cohort of 2306 men and women) investigated the associations between body composition and age-related decline in gait speed in older adults followed for 4 years and it was found that the thigh intermuscular fat is associated with physical dysfunction due to an annual gait speed decline, and this decline was intensified with intermuscular adipose gain over time [33]. These studies demonstrated that fat infiltration into muscle contributes to mobility loss with aging. Therefore, the age-related pathological effects of adipose tissue in the muscle are related to muscle quality and characterized by increases in connective tissue, lipid infiltration, and disturbed muscle metabolism. The lipid deposition in the muscle can also induce a transition of type II fibers to a type I phenotype, leading to impairments in muscle contractile capacity of both type I and type II fibers, resulting in dramatic decrease in muscle power (force and speed) [34].

The effects of physical activity in the prevention or reversion of the loss of strength and skeletal muscle mass were investigated, as well as the gain in adiposity, in older adults [35]. Isokinetic knee extensor strength and computer tomography midthigh skeletal muscle and adipose tissue cross-sectional areas were assessed in sedentary older men at baseline and following a one-year exercise intervention. At the end of the study, all subjects demonstrated a loss of muscle mass with age. However, sedentary older subjects demonstrated greater IMAT and lower muscle strength compared to subjects who underwent exercise intervention. These data underscore the inverse relationship between IMAT accumulation and muscle strength in aging and suggest that regular physical activity can prevent both the age-associated loss of muscle strength and increase in muscle adipose infiltration in older adults with moderate functional limitations.

It is well stablished in the literature that older people gain adiposity and lose muscle mass, and that adipose tissue quantity negatively affects muscle function with aging [7,22]; however, the effects of both the quantity (body fat mass) and quality of adipose tissue must be considered, since these important characteristics affect adipose tissue secretory capacity and, consequently, modulate metabolism in other peripheral tissues [36]. While redistribution occurs in all adipose depots during aging, this review focuses on abdominal subcutaneous adipose tissue as it is the largest adipose tissue depot in the human body and its overall contribution to systemic phenotypes may be greater than visceral adipose tissue (VAT), even if the magnitude of effects per unit mass is smaller.

## 3. Adipose Tissue Cell Death in Aging

Adipocytes are generated from a pool of mesenchymal stem cells or adipose-derived stem cells (ASCs) with subsequent differentiation in to mature adipocytes [37,38] (Figure 1). ASCs are mesenchymal stem cells characterized by their self-renewal ability and their capacity to differentiate into a variety of cell types, including adipocytes, osteoblasts and myocytes [39]. The most commonly reported cell surface markers to distinguish ASCs that have adipogenic potential are CD34, CD29, CD13, CD44, CD73, CD90, CD142, and CD9 [40]. Other studies have reported combinations of CD36, CD45, CD34 and CD31 [41], as well as Lin, CD29, CD34, CD24 and Sca-1 ASCs [37]. However, it is still unknown which molecular, metabolic, and endocrine profiles of adipocytes are derived from certain specific human ASC populations [39].

With age, ASCs, mature adipocytes, and other cell types within adipose tissue (e.g., immune cells, endothelial cells, fibroblasts, et al.) can undergo to irreversible arrest of cellular proliferation and subsequently cell death by apoptosis or necrosis [18,42]. Apoptosis is a regulated and controlled process, and normally occurs in response to infectious processes. Necrosis is characterized as passive, accidental cell death resulting from environmental factors such as cytotoxicity or inflammation. In adipocytes, both necrosis and apoptosis induce free fatty acids release and macrophage infiltration, forming crown-like structures (CLS) [43]. Crown-like structures are associated with activation of nuclear factor-κB (NF-κB) and secretion of multiple inflammatory factors, such as tumor necrosis factor-α (TNF-α), interleukin 1b (IL-1b) and interleukin 6 (IL-6) [44]. Moreover, during necrosis, the inflammatory response is perpetuated by rupture of the adipocyte plasma membrane, dilation of the endoplasmic reticulum and release of cell contents into the extracellular space. Therefore, adipocyte death is associated to release of several pro-inflammatory factors that impair adipose tissue function, repair, and self-renewal [44].

The inability to recruit progenitor cells to the adipogenic lineage (i.e., self-renewal) for adipose tissue expansion via hyperplasia can lead instead to hypertrophy of existing adipocytes, which is associated with metabolic inflexibility, inflammation, necrosis (presence of crown-like structures), and metabolically delirious secretory profile (i.e., adiponectin release). Recent small humans studies examining ASCs ex vivo have identified a lower capacity of ASCs to self-renew and differentiate into adipocytes in aged (~71 years) compared to young (~27 years) humans [45].

Recent evidence suggests that aging promotes decreases proliferative capacity and increases expression of senescence-related genes in human ASCs [46]. ASCs from healthy young, middle aged, and aged volunteers were compared and the aged ASCs presented higher expression of p16^INK4A^ (senescence marker) and antiapoptotic gene BCL2 and proapoptotic gene BAX (BCL2-associated X protein), upregulation of NFκB (factor nuclear kappa B), TNF-α and the genes for their corresponding receptors. Moreover, downregulation of genes associated with tissue renewal capacity such as p53 (tumor protein 53), caspase 3, caspase 8, and caspase 9 were observed, which indicates age-related impacts on ASCs proliferation and differentiation and impairment in self-renewal and repair capabilities [46].

These data suggest that impaired “adipogenic capacity”, which is the adipose-derived progenitor capacity of the expression of transcription factors necessary for cell differentiation, may contribute to pathologies of aging, such as inflammation, insulin resistance, and impaired regenerative capacity. When adipose tissue loses this ability to expand due to changes in cell or tissue structure and function, it becomes more susceptible to multiple forms of metabolic dysfunction that affect local and physiological health during aging [47].

It is possible that the age-related changes in ASCs are intrinsic (i.e., genetic) or attributed to changes in niche/environment (i.e., epigenetic). These changes have not been thoroughly explored in humans. One potential way for addressing this gap in knowledge would be transplanting ASCs from young mice into older mice and vice-versa. Given the increasing prevalence of type 2 diabetes and obesity, and the rapidly expanding aging population, animal and human studies will be critical to gaining further insight into the function of ASCs cells, and defining their role in skeletal muscle and whole-body metabolism, as well as their impact on human health.

## 4. Cellular Senescence in Adipose Tissue with Aging

Cellular senescence is a stress response process by which damaged cells exit the cell cycle permanently [48,49]. Cellular senescence serves an important tumor suppressive function by growth arresting potentially pre-neoplastic cells [49]. When cells encounter various damage signals, cell stresses, and oncogenic insults, these can trigger a senescence cascade. Initially these damaged cells can enter a reversible cell cycle arrest that halts proliferation to attempt cellular repair via p53 mediated DNA damage response. In the event that repair is impossible, then the cell can enter cell senescence and permanently exit the cell cycle, which requires expression of cyclin-dependent kinase inhibitors, notably the tumor suppressor protein p16^INK4A^ [50]. This highly metabolically active, permanent growth arrested state is further reinforced through heterochromatinization of cell-cycle genes and establishment of the senescence-associated secretory phenotype (SASP), which includes pro-inflammatory cytokines that maintain cell cycle arrest.

The SASP contributes more than autocrine signaling to maintain cell cycle arrest. The SASP consists of an array of cytokines, chemokines, proteases, growth factors, and matrix remodeling factors. SASP signaling should initiate an immune response, resulting in clearance of the irreversibly damaged cells and their potentially pre-neoplastic neighbors [51,52]. However, senescent cells are highly resistant to apoptosis, and avoid immune clearance, which contributes to a growing accumulation of senescent cells in vivo over time. Furthermore, SASP factors can act in a paracrine fashion, triggering inflammatory activation by triggering engagement of the senescent cell program in neighboring cells, (termed ‘secondary senescence’ or ‘bystander senescence’) [53,54,55]. Additionally, the SASP can contribute to a pro-inflammatory/profibrotic milieu that can not only affect the local tissue and organ environment but also spill into the circulation, leading to deleterious systemic effects and chronic sterile inflammation [52]. Nevertheless, chronic exposure to the SASP caused by the aging process can lead to tissue dysfunction [51,56]. Also, given the broad array of stressors and damage signals that induce senescence, cell type differences, and SASP factors, there is no single biomarker of cell senescence in vivo. Therefore, senescence cells are best identified by the quantification of multiple biomarkers such as p16^INK4A^ and p21 Cip1/Waf1 encoded by Cdkn2a and Cdkn1a, DNA damage foci (i.e., γH2A.X), and telomere dysfunction [57,58].

Though senescent cells can accumulate with advancing age in many organs, one tissue type in which these cells are speculated to be of considerable importance for both local and systemic dysfunction is adipose tissue [59,60]. It is important to consider that the senescent cells have not only negative but also positive roles, since this process is involved in embryogenesis and tumorigenic cells neutralization and is necessary for body health. Harmful effects occur when senescent cell abundance increases above a critical threshold, specifically with aging. In rodents, senescent cells within adipose tissue can downregulate key transcriptional factors, decrease adipogenesis, and promote adipocyte atrophy [58]. In older humans, adipocytes and immune senescent cells can infiltrate the skeletal muscle and exert detrimental effects on muscle fibers [26]. For example, recent evidence suggests an association of a marker of adipose tissue senescent cell burden, p16^INK4A^, with poor mobility as measured by endurance walk distance, usual gait speed, grip strength, short physical performance battery, and perceived mobility disability in human [33,61]. Therefore, senescent cells accumulate in adipose tissue with aging across a number of mammalian species including humans [55,59,62] and, given that adipose is frequently the largest organ in humans, this may have profound systemic consequences.

Taken together, those factors implicated in multi-system physiologic decline due to the important immune and endocrine functions of adipose tissue [59]. Since adipose tissue has a pivotal role in maintaining tissue-specific and whole-body homeostasis (including skeletal muscle glucose homeostasis and insulin responses), the implications adipose tissue on metabolic and functional changes have significant potential implications to mobility disability and promoting higher risk of disability, loss of independence, and mortality in older adults. Therefore, further investigation of the abundance and functional consequences of cellular senescence in humans is necessary to evaluate the translational potential of therapeutically targeting senescent cells and to possibly prevent physical dysfunction.

## 5. Adipose Tissue Inflammation during Aging

Adipose tissue plays an essential role in age-related metabolic dysfunction and is linked to systemic, chronic, and low-grade inflammation. Aging is associated with a decline in immune competence referred to as immunosenescence, which leads to chronic, low-grade inflammation named “inflammaging” [13]. Adipose tissue inflammation results in the release of pro-inflammatory cytokines such as TNF-α, IL-1b, IL-6 and monocyte chemoattractant protein (MCP-1/CCL2) from adipose tissue macrophages [23,24], and these factors are also associated to the presence of senescent cells (p16^INK4A^) leading to immunosenescence [13].

The exposure of macrophages to hormones, cytokines, chemokines, and fatty acids found within dysfunctional adipose tissue stimulates them to adopt a pro-inflammatory phenotype inflammation. Age-associated adipose tissue inflammation is related to the activation of the NFκB signaling pathway, with promotes a pro-inflammatory polarization of adipose tissue macrophages, independent from total adipose tissue mass [13]. The stimulus for age-associated macrophage pro-inflammatory polarization can be associated with endoplasmic reticulum stress, which promotes a pro-inflammatory environment due to the release of IL-6, MCP-1, and TNF-α cytokines within adipose tissue. The decreased vascularization promoted by aging promotes local hypoxia and favors adipose tissue macrophage accumulation [63]. The macrophage infiltration is perpetuated by a reduction in the mitochondrial cytochrome c oxidase subunit 5B (COX5B) component of complex IV within adipocytes, which inhibits lipolytic signaling and free-fatty acid mobilization and increases intracellular lipid storage [64]. Hypertrophic expansion of adipocytes promotes stress signals, thereby contributing to immunometabolic dysfunction both locally and systemically [23].

Besides macrophages, dysfunctional adipose tissue can exacerbate immunosenescence by promoting activation and differentiation of immune cells such as B cells (B lymphocytes), nautral killer (NK) lymphocytes, regulatory T cells, neutrophils, eosinophils, mast cells, dendritic cells. Immune cell accumulation in AT induces pro-inflammatory cytokine release leads to inflammasome activation and formation of crown-like structure [13]. The function of immune cells have been investigated in obesity, however some studies suggest that the pro-inflammatory microenvironment of aged murine adipose tissue, characterized by TNF-α, IFN-γ, IL-6 and prostaglandin E2 (PGE2) release, could promote NFκB activation consequently modulate dendritic cell function, also can activate neutrophils [13,65]. Controversially, the pro-inflammatory state can inhibit regulatory T cells and NK lymphocytes [13], and also impair anti-inflammatory defense.

Taken together, the chronic inflammatory environment promoted by aging can contribute to adipose tissue and skeletal muscle dysfunction and consequently leads to a feedback loop that perpetuates adipose inflammation and redistribution, impairs progenitor cell differentiation and leads to cellular dysfunction and death of adipocytes and neighboring cells such as skeletal muscle cells. The effects of inflammation on skeletal muscle is also related to the effects of inflammatory mediators on muscle protein metabolism (muscle protein breakdown and synthesis) [66]; however, way in which the inflammatory factors can induce muscle wasting and the extent to which they do so remains a largely unexplored area of research in aging.

## 6. Adipose Tissue Metabolic Flexibility and Aging

Metabolic flexibility refers to the capacity for cells and tissues to shift its substrate utilization in response to physiological stimuli such as exercise, fasting, insulin, or catecholamines [67]. Adipocyte metabolic flexibility refers to the ability of the fat cell to regulate the storage and release of fatty acids (FAs) to maintain physiological homeostasis [17]. Release of FAs from adipocytes may be regulated (homeostatic) or unregulated (dysfunctional/pathogeneic). In the former, FAs are essential since they not only serve as energy substrates but also directly and indirectly as lipid signaling molecules (lipokines). With unregulated FA release, FAs and other lipid metabolites accumulate in tissues/organs that are ill-equipped to deal with them (lipotoxicity) [26]. Over the last decade, the prevailing model for regulated intracellular FA homeostasis has been completely transformed. In this new “coupling model,” the regulated release of FAs from intracellular triacylglycerols (TAGs) by adipose triglyceride lipase (PNPLA2/PNPLA2/ATGL) is tightly coupled to mitochondrial function / lipid oxidation, as well as FA (re)esterification via peroxisome proliferator-activated receptor (PPAR) transcription factors. This coupling prevents accumulation of potential toxic FAs or FA metabolites [68]. Furthermore, the type and duration of lipolytic activation determines the relative fate of FAs (i.e., release versus oxidation). Dysregulation of this process leads to unregulated release of FAs (i.e., metabolic inflexibility) and ectopic lipid deposition, both of which can negatively impact crosstalk between adipose tissue and peripheral tissues. These aberrant changes have been suggested to be related with declines in mobility with aging [47].

Adipocytes can interact with other tissues through receptors and by the release of numerous peptides and non-peptides, which have endocrine, paracrine and systemic actions that regulate energy metabolism. Examples of such factors include adiponectin, leptin, insulin growth-like factor, lipoproteins, apolipoproteins, non-esterified fatty acids, as well as cytokines and immune-related proteins as TNF-alpha, adipsin, resistin, and interleukin 6 [36,69]. The inflammatory cytokines released by adipocytes, such as interleukins (IL-6 and IL-1b), can impact myocytes by downregulating insulin-like growth factor/protein kinase B (IGF/Akt) signaling and impair insulin signaling, compromising myoblast differentiation/proliferation capacity, and consequently decreasing muscle protein synthesis [12]. Furthermore, the impairment in insulin signaling can also disturb the muscle mass and adipose tissue metabolism by harming glucose uptake through skeletal muscle and the suppression of non-esterified free fatty acid release by the adipose tissue [17]. Adipose tissue lipid sequestration via adipocyte proliferation and differentiation—the co-called “expandability hypothesis” observed primarily in abdominal subcutaneous adipose tissue [70]—constitutes a critical defense mechanism against lipotoxicity and metabolic disease observed in aging [59,71,72,73]. The inability to recruit progenitor cells to the adipogenic lineage (i.e., self-renewal) to execute adipose tissue expansion via hyperplasia can lead instead to hypertrophy of existing adipocytes, which has been associated with metabolic inflexibility, inflammation, necrosis, and reduced adiponectin release [36,67]. Furthermore, hypertrophic adipocytes exhibit higher rates of basal lipolysis, which increases the release of free fatty acids into circulation inducing ectopic lipid accumulation and lipotoxicity [36] and accelerate the development of age-related diseases [47].

The effect of increased fatty acids release was tested in preadipocytes cultured from rats of different ages. Cells from old animals accumulated lipid in multiple small lipid droplets, undergo apoptosis, and have increased levels of apoptotic proteins such as caspase activity, BAX (BCL2-associated X protein), and p53 (tumor protein 53). In young animals, the oleic acid promoted adipogenesis in preadipocytes and increased lipid droplet formation. Oleic acid oxidation was impaired in preadipocytes and mature adipocytes from old animals. These data suggest that, with aging, preadipocyte fatty acid handling becomes dysfunctional, making these cells more susceptible to lipotoxicity and fat tissue dysfunction [74].

Therefore, there is evidence that metabolically flexible adipocytes allow for the regulated release of energy substrates, metabolites, lipokines, and/or adipokines and that alteration in adipose tissue metabolic flexibility (by age, diet, or PNPLA2/ATGL genotype) influence skeletal muscle. However, a comprehensive evaluation of the above processes and mechanisms in aging humans has not been performed.

## 7. Adipose-Muscle Crosstalk, Mobility Disability, and Aging

Aging is associated with decrements in the quality of muscle, such as loss of motor units and changes in fibers type proportion. Changes in muscle composition like increased connective tissue, fatty infiltration, and altered muscle metabolism can also be observed. All those factors could affect neuromuscular activation and physical performance, leading to functional disability [8].

The progressive decline in mobility with aging has been mostly attributed to muscle mass and quality wasting and increased fat mass, and there is an association with adipose tissue factors. Regarding contractile protein content, there is some evidence that the human adipose tissue secretome can decrease myosin heavy chain II and troponin expression in human muscle cells in vitro [12]. The metabolic disturbances caused by excess lipid storage can also change the myogenic program but modulating specific transcription factors such as myoblast determination protein (MyoD) and myogenic factor 4, which regulates the expression of contractile proteins such as myosin, tropomyosin and troponin [75], and titin, which is a protein related to maintenance of sarcomere integrity [76]. Moreover, recent study evidenced that the secretome of obese visceral adipocytes can affect myotubes through the modulation of the expression of contractile proteins such as MyoD and myogenin; by decreasing levels of growth factors such as IGF2 and IGFBP-5, modulating neuromuscular junction proteins (i.e., muscle-specific tyrosine kinase), and decreasing the levels of titin [12], which is a protein related to sarcomere structure and elasticity [77]. Therefore, human adipose tissue-secreted factors seem to be able to disrupt myotubes internal sarcomere structure and function, impair myogenic capacity of the skeletal myoblasts, and might underlie muscle wasting and mobility disability [12].

Some studies have explored the effects of aging on the adipose tissue secretome in animals [12,78]. The effects of secreted factors from visceral and subcutaneous adipose tissue on myocyte structure were investigated and revealed that myocytes cocultured with visceral adipose tissue were smaller. In addition, there was a decrease in gene expression of muscle-specific structural proteins and growth/myogenic factor IGF-2, which participates in the protein signaling pathway, consistent with a profile associated with muscle atrophy were presenting a profile indicative of muscle atrophy [7,10]. A recent study examined muscle atrophy in terms of cellular and molecular characteristics of skeletal muscle in young and aged mice fed with a high-fat diet for 12 or 24 weeks. The authors found that, in comparison with young mice, the aged animals had higher body weight, but lower gastrocnemius muscle mass. Computed tomography scan analysis showed higher ectopic fat accumulation in the skeletal muscle in the lower leg of aged mice, and a reduction in only type II muscle fibers of aged mice [78]. The researchers also showed that the aged mice presented upregulation of the expression of genes associated with inflammation (PAI-1, Il-1β, and TNF-α), cellular senescence (p16 and p19) and protein degradation (Atrogin1 and Murf1) in aged mice muscle, whereas downregulation of genes associated with differentiation and regeneration (Myogenin, Myf5 and MyoD), mitochondrial lipid metabolism (PGC-1 α, PPAR-α/δ, ACADM, ACSL-1, CPT-1α, and UCP 3), tissue fibrosis (Tgf1β and collagen3a1) and angiogenesis (Cd 31). Overall, this study demonstrates that peri-muscular adipose tissue deposition accelerates aging-induced muscle atrophy factors related to proteolysis and senescence in muscle and induce a progressive loss of skeletal muscle mass and function (sarcopenia) [78].

Besides the effects observed in myotubes and muscle fiber structure, the metabolic shifts such as higher levels of glycolysis, lower levels of fatty acid oxidation promoted by the senescence process can induce apoptosis resistance, and promote higher levels of protein synthesis and reactive oxygen species generation [51]. The critical point is that the higher levels of fatty acid can establish an imbalanced crosstalk between adipose tissue and other metabolically relevant tissues, such as muscle, since that fatty acids are not being oxidized or stored as neutral triacylglycerols, leading to further increases in fatty acids release, perpetuating adipocyte and adipose tissue dysfunction [74], pro-inflammatory profile, insulin resistance, and muscle wasting. Taken together all these factors would perpetuate the metabolic inflexibility status promoted by aging and contribute for the development of muscle atrophy and disability [25].

**Figure 2:**
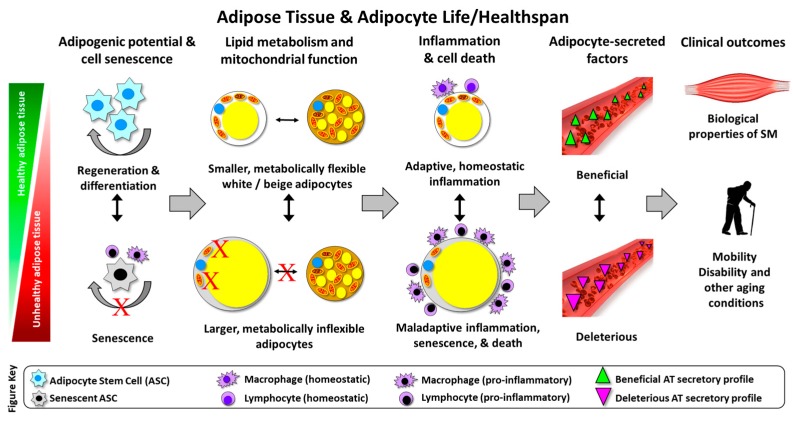
Communication from adipose tissue to muscle cells leading to muscle atrophy and mobility disability. The green side represents the healthy adipose tissue, which presents adipogenic potential and balance through adipocyte stem cells (ASCs) regeneration and differentiation leading to metabolically flexible adipocytes and beneficial-secreted factors. The red side represents the unhealthy adipose tissue and impaired “adipogenic capacity”, which shows the effects of aging in ASCs that undergo to senescence. Senescence is characterized by irreversible arrest of cellular proliferation that establish a senescence-associated secretory phenotype (SASP), leading to maladaptive inflammation and cell death, which can ultimately mediate inter-organ pathology, including skeletal muscle pathology (mobility disability).

Figure 2 summarizes the evidence for communication from adipose tissue to muscle leading to muscle atrophy and mobility disability.

## 8. Conclusions

The importance of adipose tissue to the whole-body metabolism is undeniable; however, the impact of aging on adipose tissue dynamics, as well as its cross-talk with skeletal muscle, remains unknown in the context of mobility-disability of aging. High adiposity predicts onset of mobility disability even in well-functioning older persons [79]. Adipose distribution/re-distribution in aging has been suggested to be related with declines in mobility [47], yet a comprehensive assessment of the contribution of each adipose depot to mobility has not been undertaken. The status quo for aging-related mobility disability is that the primary underlying pathological mechanism is the inability of skeletal muscle to support locomotion due to aberrations in intrinsic key biological properties of skeletal muscle that are yet to be identified. This review discussed some evidence about a potentially much more complex role for adipose tissue—not only involving adipose tissue quantity, but also quality and adipose tissue-secreted factors—in mobility disability either by directly or indirectly influencing skeletal muscle. Therefore, understanding the cellular senescence and exploring the ability of adipose tissue structural and functional properties to predict the onset of mobility disability, as well as the effects of lifestyle choices such as diet and physical activity in aging process, are necessary for the comprehension of its effects on the health span. Efforts should be continued to elucidate the adipose tissue-skeletal muscle crosstalk and aging process in order to provide the development of strategies that preserve quality of life and prevent morbidity/mortality in the aging population.
